# Muesli Intake May Protect Against Coronary Artery Disease: Mendelian Randomization on 13 Dietary Traits

**DOI:** 10.1016/j.jacadv.2024.100888

**Published:** 2024-03-06

**Authors:** Joshua K. Park, Ben Omega Petrazzini, Shantanu Bafna, Áine Duffy, Iain S. Forrest, Ha My Vy, Carla Marquez-Luna, Marie Verbanck, Jagat Narula, Robert S. Rosenson, Daniel M. Jordan, Ghislain Rocheleau, Ron Do

**Affiliations:** a Charles Bronfman Institute for Personalized Medicine, Icahn School of Medicine at Mount Sinai, New York, New York, USA;; b Department of Genetics and Genomic Sciences, Icahn School of Medicine at Mount Sinai, New York, New York, USA;; c Medical Scientist Training Program, Icahn School of Medicine at Mount Sinai, New York, New York, USA;; d UR 7537 BioSTM, Université Paris Cité, Paris, France;; e Department of Medicine, Icahn School of Medicine at Mount Sinai, New York, New York, USA;; f Cardiovascular Imaging Program, Zena and Michael A. Wiener Cardiovascular Institute, Mount Sinai Heart, Icahn School of Medicine at Mount Sinai, New York, New York, USA; and the; g Metabolism and Lipids Unit, Zena and Michael A. Wiener Cardiovascular Institute, Mount Sinai Heart, Icahn School of Medicine at Mount Sinai, New York, New York, USA.

**Keywords:** acetate, CARDIoGRAMplusC4D, cardiovascular disease, genetic epidemiology, metabolomics, UK Biobank

## Abstract

**BACKGROUND:**

Diet is a key modifiable risk factor of coronary artery disease (CAD). However, the causal effects of specific dietary traits on CAD risk remain unclear. With the expansion of dietary data in population biobanks, Mendelian randomization (MR) could help enable the efficient estimation of causality in diet-disease associations.

**OBJECTIVES:**

The primary goal was to test causality for 13 common dietary traits on CAD risk using a systematic 2-sample MR framework. A secondary goal was to identify plasma metabolites mediating diet-CAD associations suspected to be causal.

**METHODS:**

Cross-sectional genetic and dietary data on up to 420,531 UK Biobank and 184,305 CARDIoGRAMplusC4D individuals of European ancestry were used in 2-sample MR. The primary analysis used fixed effect inverse-variance weighted regression, while sensitivity analyses used weighted median estimation, MR-Egger regression, and MR-Pleiotropy Residual Sum and Outlier.

**RESULTS:**

Genetic variants serving as proxies for muesli intake were negatively associated with CAD risk (OR: 0.74; 95% CI: 0.65–0.84; *P* = 5.385 × 10^−4^). Sensitivity analyses using weighted median estimation supported this with a significant association in the same direction. Additionally, we identified higher plasma acetate levels as a potential mediator (OR: 0.03; 95% CI: 0.01–0.12; *P* = 1.15 × 10^−4^).

**CONCLUSIONS:**

Muesli, a mixture of oats, seeds, nuts, dried fruit, and milk, may causally reduce CAD risk. Circulating levels of acetate, a gut microbiota-derived short-chain fatty acid, could be mediating its cardioprotective effects. These findings highlight the role of gut flora in cardiovascular health and help prioritize randomized trials on dietary interventions for CAD.

Coronary artery disease (CAD) is a leading cause of global death, accounting for 9.4 million deaths each year.^1^ Diet is a modifiable lifestyle factor amenable to safe, early, and cost-effective intervention.^2^ Previously, the INTERHEART study, a large, international, case-control study assessing the importance of risk factors for coronary artery disease worldwide, attributed 30% of CAD risk to poor dietary habits.^3^ Meanwhile, the CORDIOPREV study (Coronary Diet Intervention With Olive Oil and Cardiovascular Prevention)^4^ and the Lyon Diet Heart Study^5^ highlighted the protective effects of a Mediterranean diet in secondary cardiovascular disease prevention. Dietary interventions have also demonstrated a 45% reduction in CAD-related mortality^6^ and signs of reversing atherosclerosis.^7^ While past studies have examined certain foods, such as red meat,^8^ fish,^9^ and plant oils,^10^ the full diversity of foods and beverages in the human diet remains to be tested for causal effects on CAD risk.

Causal relationships between diet and CAD remain unclear because observational cohort studies carry limitations preventing causal interpretations. Depending on their design, some observational studies may be biased by residual confounders, which can encompass a range of lifestyle factors, such as smoking and physical activity, as well as environmental determinants like socioeconomic status, toxin exposure, and health care access. Reverse causation is another potential bias, where individuals alter their diet in response to their condition rather than the diet causing the condition. These biases may explain why several prominent observational findings were later contradicted by evidence from randomized controlled trials (RCTs).^11,12^ RCTs provide a critical approach to addressing some of these challenges, but they too are susceptible to error.^13^ Additionally, RCTs face ethical, financial, and methodological constraints, including stringent participant selection criteria. To ascertain causality in diet-CAD associations, an integrated approach combining multiple study designs is needed to “triangulate”^14^ and consolidate the evidence from multiple approaches.

Mendelian randomization (MR) is a method employed in genetic epidemiology that leverages genetic variants as instrumental variables (IVs) to infer, under critical assumptions, causal links in observational data between modifiable exposures (such as risk factors) and outcomes of interest (disease).^15^ By utilizing germline variants, in which alleles are randomly allocated at conception prior to the onset of diseases like CAD, MR emulates the random assignment characteristic of RCTs. Historically, MR faced challenges with testing dietary traits due to limited genetic data on diet, resulting in a shortage of genetic instruments representing these traits. However, with the expansion of large-scale population biobanks like the UK Biobank (UKB), there is a proliferation of dietary data linked to both genetics and health records. The unprecedented size and granularity of these data sets is enabling genome-wide association studies (GWAS) to identify potential genetic instruments for detailed dietary traits, despite the typically low genetic heritability of such variables.^16^

Here, we perform 2-sample MR to estimate the causal effects of 13 dietary traits on CAD risk. For suspected causal associations, we performed secondary MR analyses testing 171 plasma metabolites as potential mediators of those associations.

## METHODS

### ETHICAL APPROVAL.

UKB received approval from the North West Multi Centre Research Ethics Committee as a Research Tissue Bank (11/NW/0382). This study was approved by UKB application number 16218 and adheres to the principles of the Declaration of Helsinki.

### STUDY DESIGN AND PARTICIPANTS.

**FIGURE 1:**
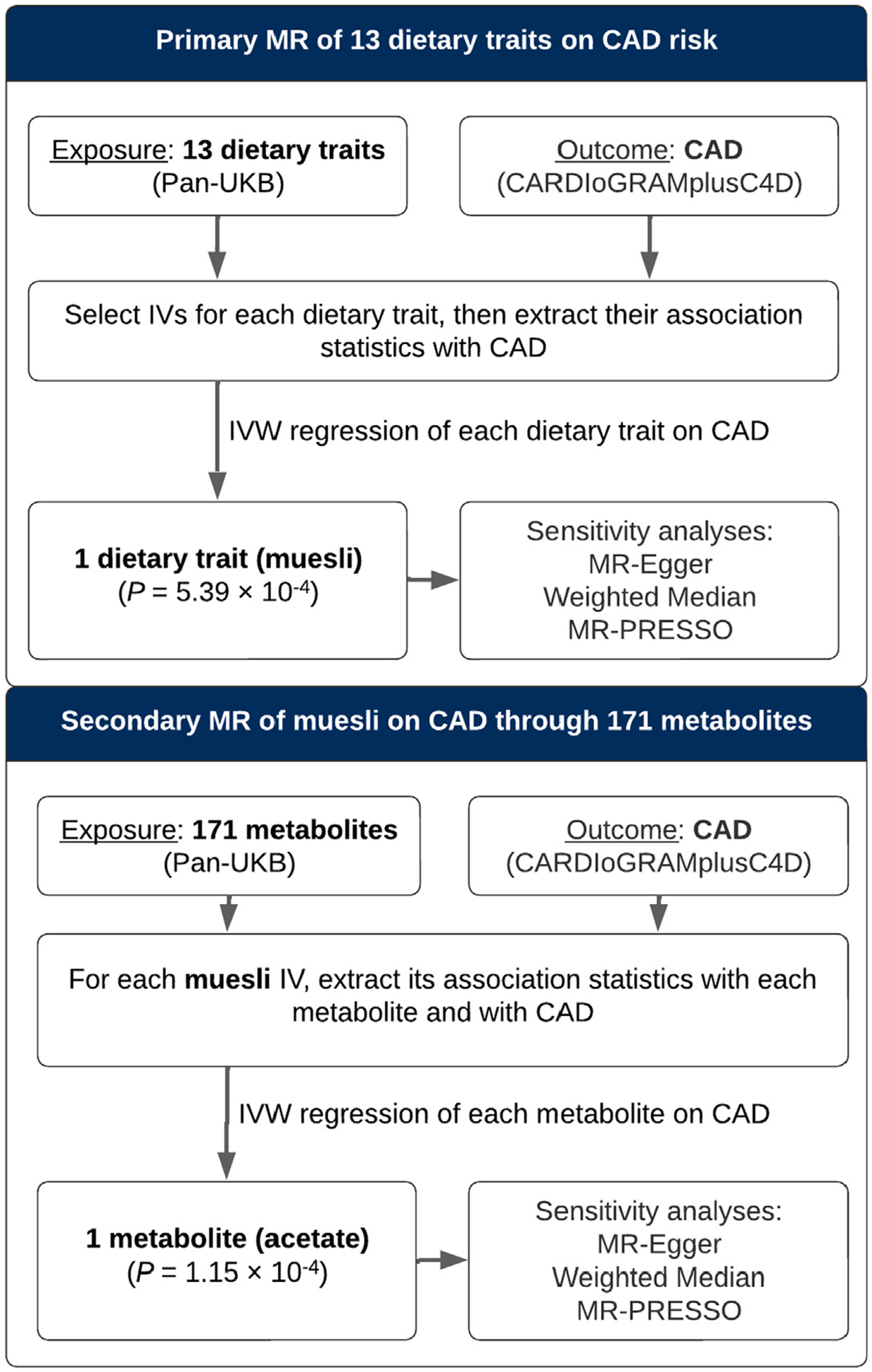
Flowchart Summarizing the Study Design CAD = coronary artery disease; CARDIoGRAMplusC4D = Coronary Artery Disease Genome-wide Replication and Meta-Analysis (CARDIoGRAM) Plus The Coronary Artery Disease (C4D) Genetics Consortium; GWAS = genome-wide association study; IV = instrumental variable; IVW regression = inverse-variance weighted regression; MR = Mendelian randomization; MR-Egger = MR-Egger regression; MR-PRESSO = Mendelian Randomization Pleiotropy Residual Sum and Outlier; Pan-UKB = Pan-Ancestry Genetic analysis of the UK Biobank; SNP = single-nucleotide polymorphism; UKB = UK Biobank.

The study design is summarized in Figure 1. Following the Strengthening the Reporting of Observational Studies in Epidemiology using MR (STROBE-MR) guidelines,^17^ we note that MR relies on 3 assumptions regarding IVs: 1) the genetic variant (eg, a single-nucleotide polymorphism [SNP]) is directly associated with the exposure (muesli intake); 2) the genetic variant is unrelated to confounders between the exposure and the outcome (CAD risk); and 3) the genetic variant has no effect on the outcome (CAD risk) other than through the exposure (muesli intake).^15^

Summary statistics were obtained on up to 429,531 unrelated, male and female individuals of European genetic ancestry in UKB, a longitudinal, population-based cohort study with genetic and phenotypic data on over 500,000 participants aged 40 to 69 years at recruitment from across the United Kingdom during 2006 to 2010.^18^ Our study also included summary statistics from the CARDioGRAMplusC4D 1000 genomes-based GWAS, which included 60,801 CAD cases and 123,504 controls.^19^ We restricted our analyses to participants of European genetic ancestry to reduce heterogeneity.

### DIETARY TRAIT ASSESSMENT.

UKB collected dietary intake information via 2 well-validated methods: 1) a touchscreen questionnaire at recruitment (2006-),^20^ which asked 29 questions related to the average frequency of consumption of common foods and food groups over the past year; and 2) a more detailed, web-based dietary recall questionnaire (2011-), which documented self-reported intake of over 200 commonly consumed foods and beverages during the preceding 24 hours.^21^ Thus, self-reported muesli intake was documented by 2 traits: one trait (data-field 1468) was of nominal categorical data collected in the touchscreen questionnaire by a multiple-choice question: “What type of cereal do you mainly eat?” The answer choices were “Bran cereal (eg, All Bran, Branflakes)”; “Biscuit cereal (eg, Weetabix)”; “Oat cereal (eg, Ready Brek, porridge)”; “Muesli”; “Other (eg, Cornflakes, Frosties)”; “Do not know”; and “Prefer not to answer”. A second trait (data-field 100800) was of ordinal categorical data collected in the 24-hour recall questionnaire by a multiple-choice question: “How many bowls of muesli?” This question was only asked to participants who reported consuming breakfast cereals the previous day. Answer choices were “none,” “1/2,” “1,” and “2+.”

### GENETIC INSTRUMENTS FOR DIETARY TRAITS.

We identified 13 dietary traits (11 nominal categorical and 2 ordinal categorical traits) with sufficient numbers of IVs (ie, >3 associated variants) in Pan-UKB for the construction of robust genetic instruments. Pan-UKB had performed 16,131 GWAS analyses on 7,221 phenotypes in ~420,531 UKB participants of European genetic ancestry.^22^ The exact sample size of each dietary trait GWAS in Pan-UKB is included in Supplemental Table 1. Using the Genome-wide Complex Trait Analysis (GCTA) software package,^23^ we performed a conditional and joint association analysis (COJO)^24^ to select independently associated SNPs for each of the 13 traits of interest in the European ancestry subset of Pan-UKB, based on the following options: -cojo-slct-cojo-p 5 × 10–8 -cojo-collinear 0.9 -cojo-wind 10,000 -diff-freq 0.2. A subset of Pan-UKB was used as the reference sample (sample size = 10,000 individuals; 17,091,093 SNPs). The 13 SNPs instrumenting muesli intake (data-field 1468) are detailed in Supplemental Table 2. The number of SNPs instrumenting each trait is also included in Supplemental Table 1.

### GENETIC INSTRUMENTS FOR CAD RISK.

We used GWAS summary statistics from the Coronary Artery Disease Genome-wide Replication and Meta-analysis plus The Coronary Artery Disease Genetics (CARDIo-GRAMplusC4D) consortium. CARDioGRAMplusC4D (60,801 cases; 123,504 controls) combined data from 48 genetic studies to identify risk loci for CAD and myocardial infarction.^25^ We performed COJO analysis using GCTA software, using the same parameters and reference sample, as described above.

### GENETIC INSTRUMENTS FOR PLASMA METABOLITE LEVELS.

UKB recently released metabolomic data on 121,731 participants. Nightingale Health Ltd had assayed a panel of 249 metabolic biomarkers (168 in absolute values and 81 ratio measures) from plasma obtained at recruitment using nuclear magnetic resonance spectroscopy between June 2019 and April 2020. Most biomarkers were measured in concentration units (mmol/L), but exceptions are indicated in Supplemental Table 3. Details of the metabolic profiling platform have been described.^26^

To identify genetic instruments for plasma metabolite levels, we conducted genome-wide association tests on each of 171 metabolic traits, using directly genotyped or imputed genetic data as predictors, while adjusting for age, sex, and principal components 1 to 10. We then used REGENIE,^27^ a machine learning method for fitting a whole-genome regression model for quantitative and binary phenotypes that is computationally efficient. Using PLINK,^28^ a free, open-source whole-genome association analysis toolset, we then extracted summary statistics for the 13 SNPS instrumenting muesli intake listed in Supplemental Table 2.

### STATISTICAL ANALYSES.

Analyses were performed using R 4.1.0.^29^ The primary analysis used fixed effect, inverse-variance weighted (IVW) regression.^30^ MR-Egger regression^31^ and weighted median estimation^32^ were also used in sensitivity analyses to detect signs of IV assumption violations, namely horizontal pleiotropy. The trait “smoking status: never” was included in this analysis as a control, and a Bonferroni-adjusted threshold for statistical significance was used for 13 dietary traits and 1 control trait: *P* < 0.05/14 = 3.571 × 10^−3^.

To detect IVs sensitive to horizontal pleiotropy, MR-Pleiotropy Residual Sum and Outlier (PRESSO) outlier tests^33^ were conducted on 9 dietary traits that had significant MR-PRESSO global test results (*P* < 0.05) in the primary analysis. Between 1 and 2 SNPs were identified as outliers and removed for 6 of the 9 traits based on a corrected significance threshold (*P* < 0.05/n), where n was the number of SNPs instrumenting each trait. IVW tests were then rerun without the outlier IVs for these 6 traits. No SNPs were removed for the remaining 3 traits that had nonsignificant MR-PRESSO global test results.

In addition to recording muesli intake as a nominal categorical trait (data-field 1468), UKB had documented the amount of muesli consumed as an ordinal categorical trait (data-field 100800). However, this ordinal trait did not have sufficient genetic association data to construct instruments in the primary IVW analysis. We thus used IVs for the nominal trait (data-field 1468) as exposure instruments and extracted association statistics with the ordinal trait (data-field 100800) for MR analysis, as described previously. Statistical significance was defined conventionally for this follow-up analysis (*P* < 0.05).

Since diet can influence the plasma metabolome and metabolites can suggest biological mechanisms, we further tested whether the effects of muesli on CAD could be mediated by any of the 171 plasma metabolites measured in UKB. The IVs for muesli intake served as exposure instruments (Supplemental Table 2), and for each IV, association statistics with each metabolite were extracted. Each allele was then harmonized to ensure consistency between the effect and non-effect alleles. Subsequently, we performed MR using IVW, MR-Egger, and weighted median methods with a Bonferroni-corrected threshold for statistical significance (*P* < 0.05/171 = 2.923 × 10^−4^).

### DATA AVAILABILITY.

Data generated in this study are included in this paper and in the Supplemental Tables. All analyses used publicly available data from UKB.

## RESULTS

### GENETIC VARIANTS SERVING AS PROXIES FOR MUESLI INTAKE INVERSELY ASSOCIATES WITH CAD RISK.

**FIGURE 2:**
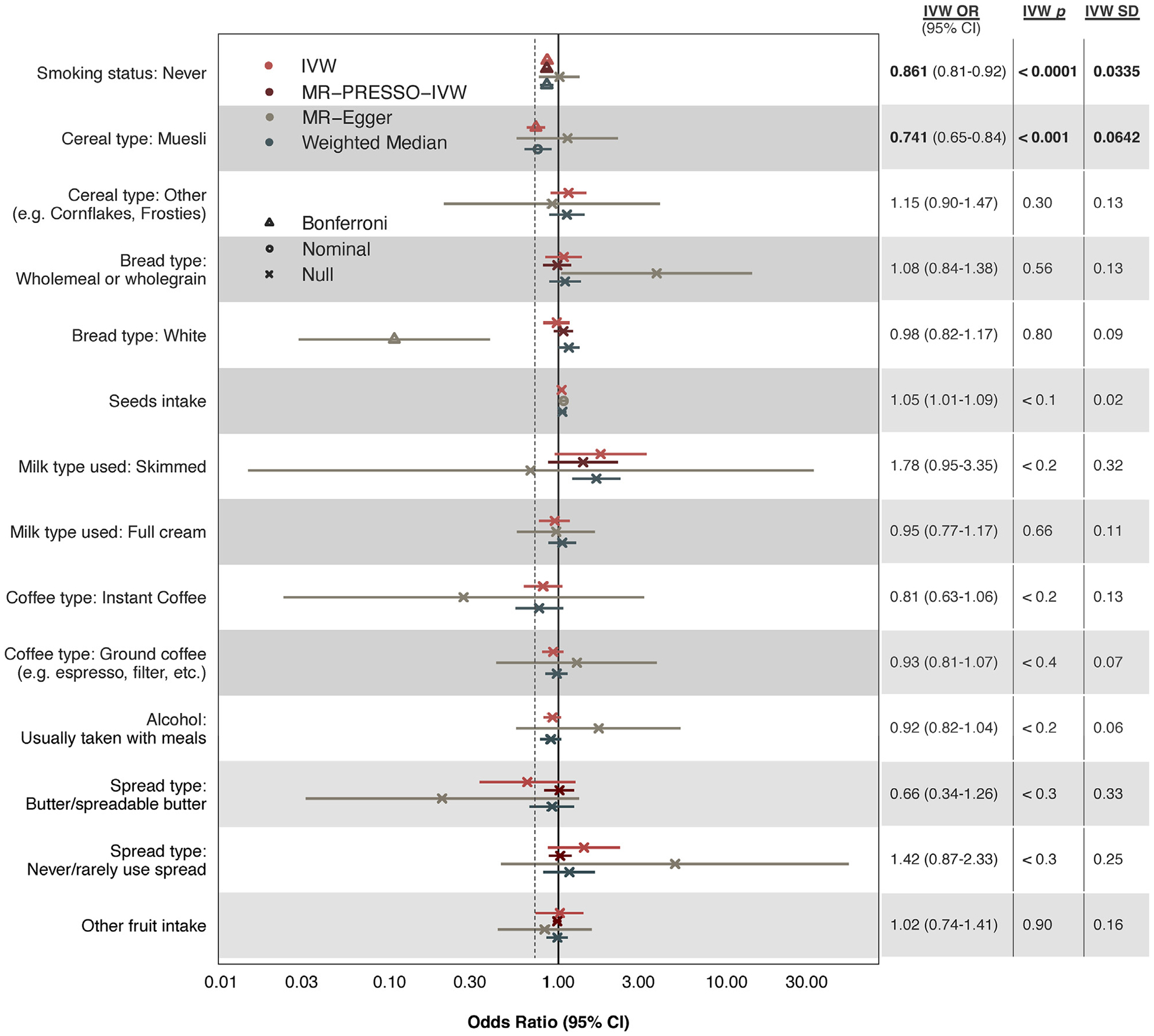
Causal Estimates of 13 Genetically Proxied Dietary Traits on CAD Risk These analyses used nominal categorical data on self-reported dietary intake in the past year. ORs represent the likelihood of CAD in individuals who report consuming the dietary item compared to those who do not. A genetically proxied dietary trait refers to a dietary pattern that is estimated using genetic variants associated with that trait as instrumental variables (ie, proxies). The analysis includes various methods: inverse-variance weighting (IVW), IVW regression after MR-PRESSO outlier test and removal of outlier instruments, MR-Egger, and the Weighted Median approach. Error bars represent 95% CIs for each estimate. The dashed vertical line reflects the finding of interest. Null corresponds to *P* > 0.05; nominal to 3.57 × 10^−3^ < *P* < 0.05; and Bonferroni to *P* < 3.57 × 10^−3^. IVW *p* = *P* value associated with the IVW test; IVW SD = standard deviation associated with the IVW test; MR = Mendelian randomization; MR-PRESSO = Mendelian Randomization Pleiotropy Residual Sum and Outlier.

In primary analyses using IVW regression, the control trait “smoking status: never” was negatively associated with CAD risk (OR: 0.86; 95% CI: 0.81–0.92; *P* = 1.626 × 10^−5^), as expected. Notably, 1 dietary trait (muesli intake; data-field 1468) was significantly and negatively associated with CAD risk (OR: 0.74; 95% CI: 0.65–0.84; *P* = 5.385 × 10^−4^) (Figure 2). Primary analysis of all other dietary traits using IVW regression was inconclusive, as they did not reach a conservative Bonferroni-adjusted threshold for statistical significance.

In sensitivity analyses, we used several MR methods robust to horizontal pleiotropy and/or account for horizontal pleiotropy. With weighted median estimation, we observed a Bonferroni-significant, negative association between the control trait and CAD risk (OR: 0.86; 95% CI: 0.79–0.93; *P* = 6.544 × 10^−4^). Muesli intake was again associated with reduced CAD risk, although this association was of nominal statistical significance (OR: 0.76; 95% CI: 0.63–0.91; *P* = 1.117 × 10^−2^) (Figure 2). MR-Egger did not detect significant associations between CAD and muesli (OR: 1.13; 95% CI: 0.57–2.26; *P* = 0.727) or the control (OR: 1.01; 95% CI: 0.77–1.33; *P* = 0.916). Although MR-PRESSO global tests detected outlier SNPs for 6 traits, adjustment with MR-PRESSO outlier tests did not uncover significant associations. Full results, including the number of genetic instruments for each trait, are provided in Supplemental Table 1. Additional MR analyses testing for mediation through muesli’s ingredients suggested the potential role of dried fruits, nuts, and cholesterol-lowering milk in muesli’s cardioprotective effects (Supplemental Table 4). Importantly, these data do not provide evidence for the absence of contributions from muesli’s other ingredients.

### VALIDATION OF MUESLI CAD RISK ASSOCIATION USING ORDINAL CATEGORICAL DATA.

**FIGURE 3:**
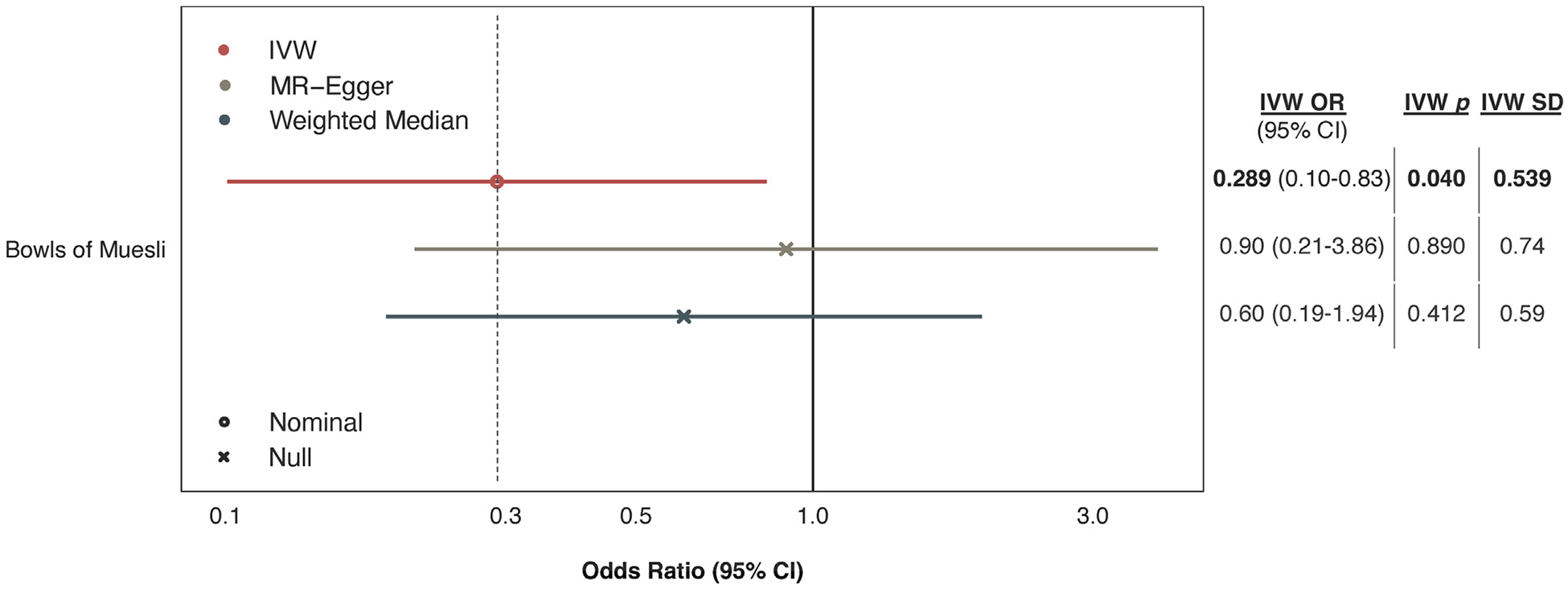
Causal Estimates of Muesli Intake Documented by 24-Hour Recall on CAD Risk These analyses used ordinal categorical data on self-reported muesli intake (data-field 100800) in the past 24 hours. Estimates are shown as ORs for coronary artery disease (CAD) risk per 1-SD increase in the consumption of bowls of muesli, for which the ordinal levels were “none”, “1/2”, “1”, and “2+”. Error bars represent 95% CIs. Estimates are derived using 3 MR methods: Inverse-variance weighting (IVW), MR-Egger, and the Weighted Median approach. Nominal statistical significance corresponds to *P* < 0.05. MR = Mendelian randomization.

UKB documented muesli intake using 2 different assessment methods, resulting in 2 different data sets on muesli: nominal categorical data (data-field 1468) and ordinal categorical data (data-field 100800). Our finding of an association between muesli and CAD risk was based on nominal data, but ordinal data can potentially provide a more graded measure of the exposure and capture subtle changes that could be masked when data are categorized without hierarchy. Thus, we also performed MR analyses between ordinal data on muesli intake and CAD risk. Supporting the primary finding, IVW regression estimated a significant negative association between the number of bowls of muesli consumed and CAD risk (OR: 0.29; 95% CI: 0.10–0.83; *P* = 0.040; Figure 3). Estimates were not statistically significant in sensitivity analyses, but the direction of effect remained consistent (Supplemental Table 5).

### PLASMA ACETATE LEVELS MAY MEDIATE THE CARDIOPROTECTIVE EFFECTS OF MUESLI INTAKE.

**FIGURE 4:**
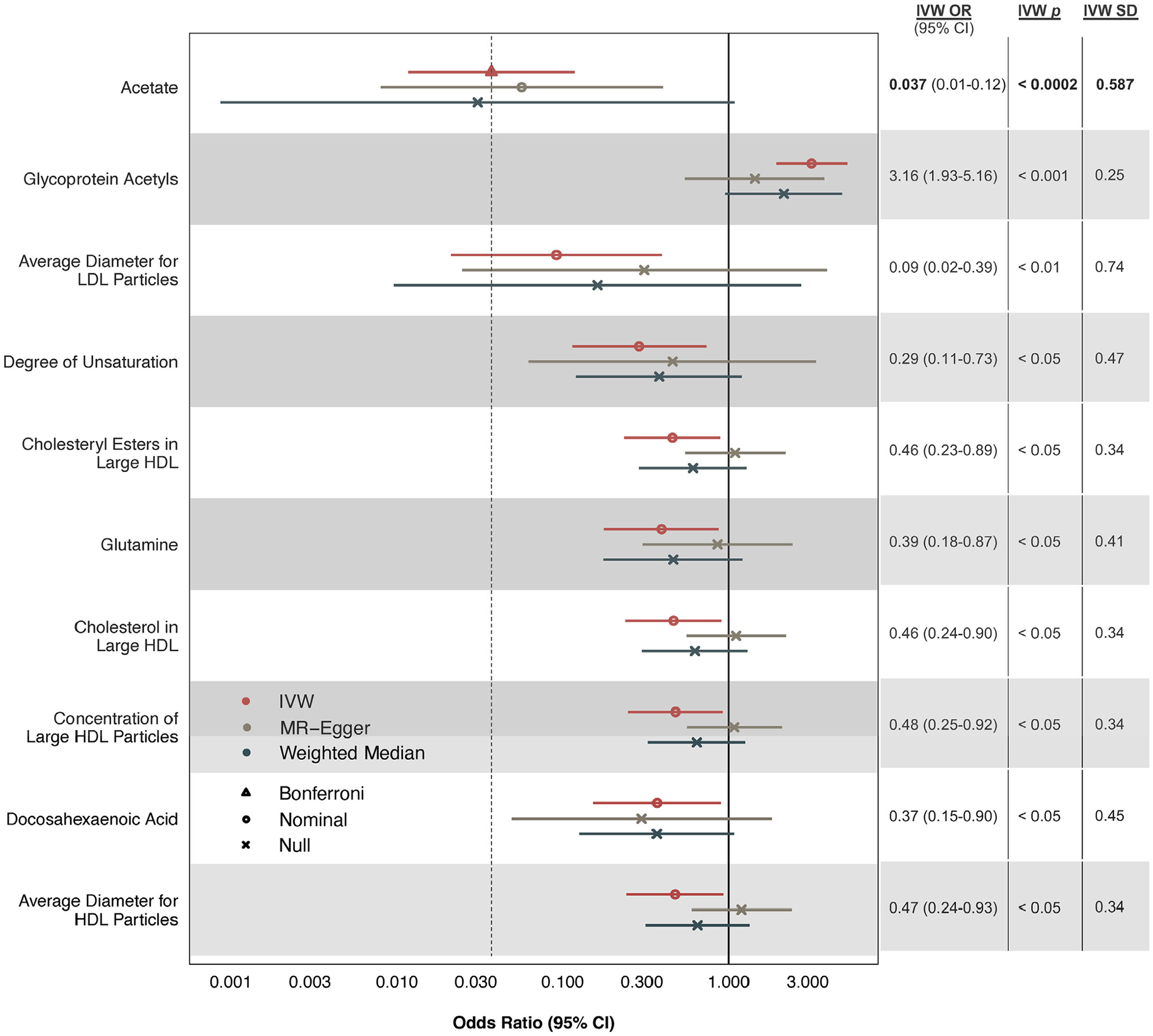
Causal Estimates of Muesli on CAD Risk Through Plasma Metabolites Causal estimates are shown as ORs per 1-SD increase in respective unit. Sample sizes and units for each measurement are also defined in Supplemental Table 3. Only 10 of 171 MR tests using IVW regression are shown here, prioritized based on statistical significance. Error bars indicate 95% CIs. Null corresponds to *P* > 0.05; nominal to 2.92 × 10^−4^ < *P* < 0.05; and Bonferroni to *P* < 2.92 × 10^−4^. Detailed results on all metabolites are provided in Supplemental Table 3. CAD = coronary artery disease; HDL = high-density lipoprotein; IVW = inverse-variance weighted; Large HDL = average diameter 12.1 nm; LDL = low-density lipoprotein; MR = Mendelian randomization.

To examine the mechanisms by which muesli intake could be reducing CAD risk, we performed secondary MR analyses testing whether the cardioprotective effects of muesli on CAD are mediated by any of the 171 plasma metabolites measured in UKB. The most significant results are shown in Figure 4, while results on all 171 plasma metabolites are provided in Supplemental Table 3. IVW regression identified 1 metabolite reaching statistical significance after Bonferroni correction for multiple testing: acetate (OR: 0.037; 95% CI: 0.01–0.12; *P* = 1.151 × 10^−4^). Sensitivity analyses using MR-Egger resulted in a significant and consistent estimate for acetate. Additionally, multivariable MR analyses accounting for plasma acetate and/or low-density lipoprotein cholesterol (LDL-C) levels suggested that increased acetate levels, rather than decreased LDL-C levels, may be mediating the protective effects of muesli (Supplemental Table 6).

## DISCUSSION

We used 2-sample MR in UKB and CARDIo-GRAMplusC4D to test for independent causal associations between 13 dietary traits and CAD risk (Central Illustration). Our results suggested genetic evidence for a potential causal relationship between muesli intake and reduced CAD risk (Figure 2). This was supported using 2 different data sets on muesli intake obtained either by food-frequency questionnaires or 24-hour recall (Figure 3). Secondary MR analyses on plasma metabolite levels suggested this may be mediated by increased levels of acetate, a gut microbiota-derived short-chain fatty acid (SCFA). These genetic data are consistent with previous observational findings of a negative association between muesli and heart disease.^34^ They also substantiate previous studies associating acetate with antihypertensive^35^ and immunoregulatory mechanisms^36^ (Figure 4). Lastly, they agree with the current guidelines for primary prevention of cardiovascular disease, which emphasizes a diet rich in fruits, vegetables, and whole grains.^37^

Muesli is commonly defined as a mixture of oats, seeds, nuts, dried fruits, and milk. Many of these nutrient-dense ingredients have been linked to cardiovascular health, suggesting biological relevance.^38,39^ Oats are notably high in a soluble fiber called β-glucan, which has been shown to reduce LDL cholesterol levels and improve insulin sensitivity.^40,41^ Oats also contain antioxidant avenanthramides, which are not present in other cereal grains, and these can support endothelial function and reduce blood pressure.^42^ Meanwhile, nuts and seeds are rich sources of omega (ω)-3 fatty acids, dietary fiber, and antioxidants, such as lignans. They are also associated with reductions in blood pressure, inflammation, and LDL cholesterol.^43,44^ Dried fruits offer essential vitamins, minerals, fibers, and antioxidant flavonoids that can further promote cardiovascular health.^45,46^ Muesli’s cardioprotective effects likely arise from a combination of mechanisms, but targeted analyses isolating key ingredients may be a useful direction for clinical translation.

Acetate is a SCFA primarily generated by colonic bacteria from dietary fiber fermentation. Increasingly, data suggest that gut microbiota-derived metabolites can modify cardiovascular health by mediating diet-microbiome-disease pathways.^47,48^ For example, dietary red meat increases systemic levels of trimethylamine-N-oxide (TMAO), a gut microbiota-generated metabolite with proatherogenic mechanisms,^49^ by providing dietary precursors, increasing microbial TMAO production, and decreasing renal TMAO excretion.^50^ In this study, we found that higher plasma acetate levels, another gut-derived metabolite, could link muesli consumption to reduced CAD risk (Figure 4). Previous studies have shown acetate can independently prevent the development of hypertension in mice and promote the colonic accumulation of anti-inflammatory regulatory T cells.^35,36^ One possibility is a potential protective muesli-acetate-CAD pathway, analogous to an atherogenic red meat-TMAO-CAD pathway.

The study had limitations. First, the validity of MR hinges on several core assumptions: namely, that genetic instruments (SNPs) are: 1) robustly associated with the exposure (muesli); 2) not confounded by common causes with the outcome (CAD); and 3) have effects on the outcome (CAD) solely through the exposure (muesli).^51^ However, the interpretation of genetic instruments for predominantly environmental traits, such as dietary intake, is complex. As Cole et al^52^ noted, dietary habits are intrinsically intertwined with each other and with non-dietary factors, potentially conflating a dietary phenotype with broader lifestyle patterns, including socioeconomic status. Moreover, genetic pleiotropy stresses the validity of 1) and 2).^53^ This study attempted to mitigate the influence of horizontal pleiotropy by conducting sensitivity analyses using multiple methods robust to horizontal pleiotropy and/or account for horizontal pleiotropy, relaxing the pleiotropy assumption.^33^ Nevertheless, causal interpretations warrant caution until the ‘triangulation’ of supportive evidence from alternative study designs, namely RCTs. A second limitation of the study is that external validity could not be tested due to the absence of genetics-linked data on muesli outside UKB. Moreover, several major dietary traits, such as red meat and egg intake, could not be tested due to the lack of robust genetic instruments for these traits. Achieving sufficient statistical power remains challenging for dietary traits, depending on the trait and its underlying genetic complexity. Furthermore, we caution against the interpretation of the study’s nonsignificant inconclusive results as evidence against a causal relationship. A third limitation relates to the dietary assessment methods used by UKB, which could be biased by recall error. We cite studies validating these methods.^54–57^ Fourth, UKB is predominantly of European genetic ancestry. Potential bias from incomplete sampling underscores the need for genetics-linked dietary data sets that encompass the entire spectrum of social and ancestral backgrounds. Cross-ancestry validation remains a priority.

## CONCLUSIONS

This study presents genetic data suggesting a causal association between the genetic instruments for muesli intake and decreased CAD risk, which may be mediated by plasma acetate levels. Additional studies are needed to ‘triangulate’ evidence from multiple study designs for causal conclusions. These data may inform randomized trials, experimental studies, and dietary guidelines.

## Supplementary Material

1

## Figures and Tables

**CENTRAL ILLUSTRATION:**
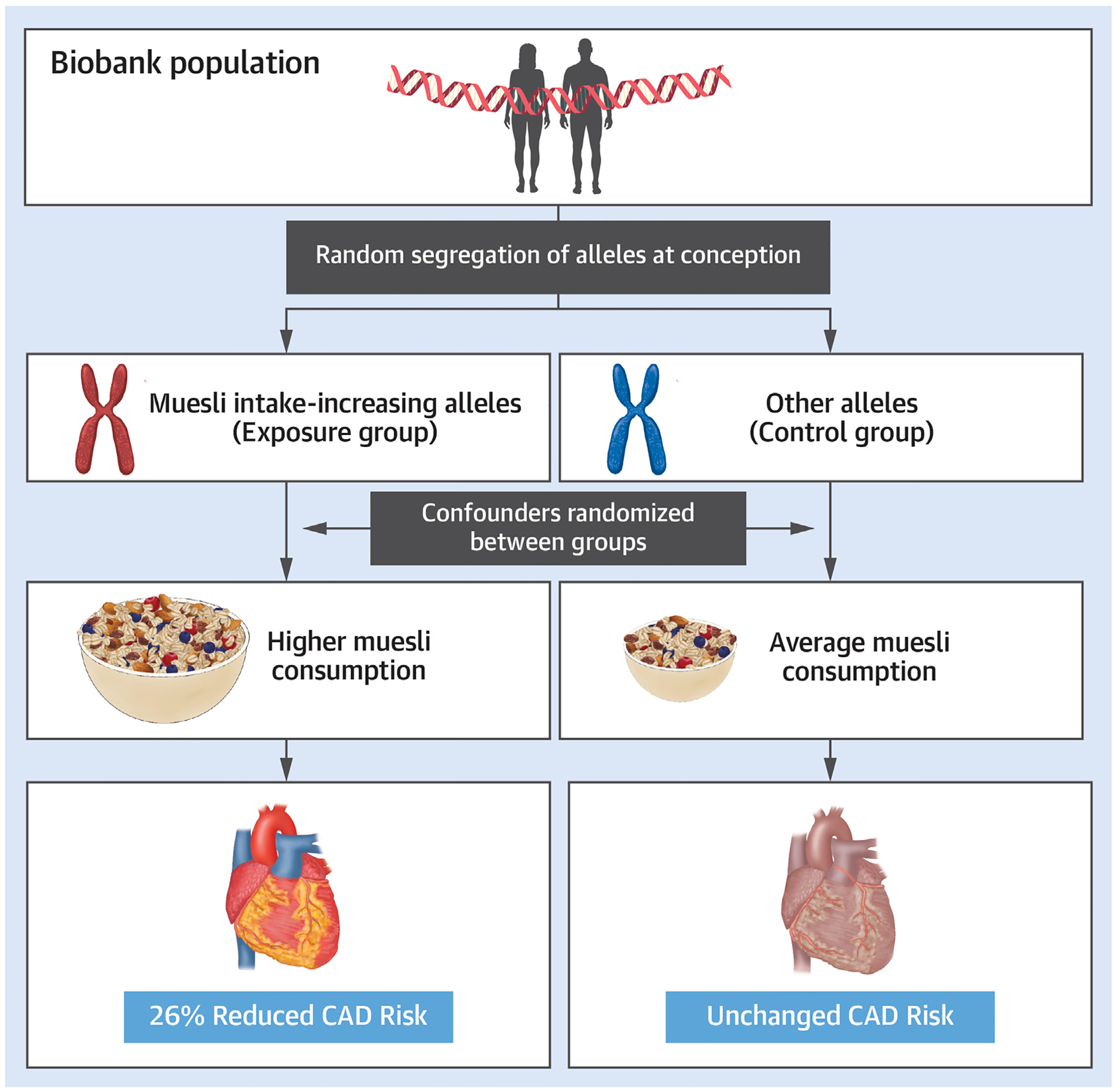
Mendelian Randomization on Muesli and Coronary Artery Disease Two-sample Mendelian randomization was performed to estimate independent causal associations between 13 dietary traits and coronary artery disease (CAD) risk. Genetic variants serving as proxies for muesli intake were significantly associated with lower CAD risk. Shown is the reduction percentage in the odds of CAD among individuals who documented consuming muesli as a nominal categorical trait compared to those who did not. 95% CI: 0.65–0.84 and *P* = 5.39 × 10^−4^.

## PERSPECTIVES

### COMPETENCY IN MEDICAL KNOWLEDGE:

Genetic data suggest that consuming muesli, a mixture of oats, nuts, seeds, and dried fruits, may causally protect against CAD risk, emphasizing the importance of a healthy, plant-based diet in cardiovascular health.

### COMPETENCY IN PATIENT CARE:

Dietary behaviors are a lifelong determinant of CAD risk. Additional studies are needed, but dietary muesli could potentially be a safe and cost-effective component in diet-based interventions for CAD.

### TRANSLATIONAL OUTLOOK 1:

Although MR is a powerful and useful method, whether muesli intake can truly cause a reduction in CAD risk must be validated by a triangulation of evidence from complementary study designs, such as animal studies and randomized trials.

### TRANSLATIONAL OUTLOOK 2:

Gut microbiota-derived SCFAs, such as acetate, are increasingly associated with pivotal roles in cardiovascular health. A better understanding of how muesli could be cardioprotective through plasma acetate is needed to potentially harness those mechanisms for therapeutic effect.

## ABBREVIATIONS AND ACRONYMS

CAD: coronary artery disease
GWAS: genome-wide association study/studies
IV: instrumental variable
IVW: inverse-variance weighted
LDL: low-density lipoprotein
MR: Mendelian randomization
OR: odds ratio
RCT: randomized controlled trial
SCFA: short-chain fatty acid
SNP: single-nucleotide polymorphism
TMAO: trimethylamine-N-oxide
UKB: United Kingdom Biobank
