# Supplementary material for: Muesli Intake May Protect Against Coronary Artery Disease: Mendelian Randomization on 13 Dietary Traits
Source: JACC Adv. Author manuscript; Available in PMC 2024 May 10. (PMC11087059; doi:10.1016/j.jacadv.2024.100888)
Supplement: 1 [file NIHMS1989104-supplement-1.docx]

**Supplemental Table 1. Causal estimates between 13 genetically proxied dietary traits and CAD risk.**

| **Trait** | **Estimate** | **SD** | **P** | **Method** | **nsnps** | **removed_nsnps** | **n_cases** | **n_controls** |
| --- | --- | --- | --- | --- | --- | --- | --- | --- |
| Smoking status: Never | -0.150191018 | 0.033482137 | 1.63E-05 | IVW | 126 | 2 | 227144 | 191673 |
| Cereal type: Muesli | -0.299979008 | 0.064191937 | 0.000538562 | IVW | 13 | 0 | 70931 | 278596 |
| Cereal type: Other, e.g. Cornflakes, Frosties | 0.138408466 | 0.12501278 | 0.300403266 | IVW | 9 | 0 | 68111 | 281416 |
| Bread type: Wholemeal or wholegrain | 0.074745451 | 0.125880588 | 0.559662542 | IVW | 20 | 1 | 233278 | 172429 |
| Bread type: White | -0.023239477 | 0.090069217 | 0.797862382 | IVW | 37 | 2 | 107422 | 298285 |
| Seeds intake | 0.044658535 | 0.019523346 | 0.070875501 | IVW | 6 | 0 | 58890 | - |
| Milk type used: Skimmed | 0.578085096 | 0.321215599 | 0.169734413 | IVW | 4 | 1 | 86430 | 333660 |
| Milk type used: Full cream | -0.050574896 | 0.106079667 | 0.65840795 | IVW | 5 | 0 | 26497 | 393593 |
| Coffee type: Instant coffee | -0.204662559 | 0.132586247 | 0.197556787 | IVW | 5 | 0 | 185482 | 144189 |
| Coffee type: Ground coffee, including espresso, filter, etc. | -0.071970369 | 0.072473704 | 0.331016479 | IVW | 24 | 0 | 73906 | 255765 |
| Alcohol usually taken with meals | -0.0809522 | 0.059441668 | 0.187668826 | IVW | 22 | 0 | 143760 | 70463 |
| Spread type: Butter/spreadable butter | -0.418435994 | 0.332488206 | 0.228794501 | IVW | 15 | 2 | 152410 | 267091 |
| Spread type: Never/rarely use spread | 0.350365178 | 0.252065156 | 0.258717608 | IVW | 4 | 1 | 42617 | 376884 |
| Other fruit intake | 0.020104184 | 0.164845862 | 0.908814228 | IVW | 5 | 1 | 58890 | - |
| Smoking status: Never | 0.014647563 | 0.139594667 | 0.916601493 | MR-Egger | 126 | 2 | 227144 | 191673 |
| Cereal type: Muesli | 0.125428984 | 0.351402215 | 0.727890926 | MR-Egger | 13 | 0 | 70931 | 278596 |
| Cereal type: Other, e.g. Cornflakes, Frosties | -0.084490179 | 0.753220544 | 0.913836001 | MR-Egger | 9 | 0 | 68111 | 281416 |
| Bread type: Wholemeal or wholegrain | 1.344985303 | 0.666673029 | 0.058810278 | MR-Egger | 20 | 1 | 233278 | 172429 |
| Bread type: White | -2.238923828 | 0.668719417 | 0.001956726 | MR-Egger | 37 | 2 | 107422 | 298285 |
| Seeds intake | 0.072137598 | 0.018978669 | 0.019087515 | MR-Egger | 6 | 0 | 58890 | - |
| Milk type used: Skimmed | -0.373275281 | 1.974360295 | 0.867492269 | MR-Egger | 4 | 1 | 86430 | 333660 |
| Milk type used: Full cream | -0.030404303 | 0.270365462 | 0.917564223 | MR-Egger | 5 | 0 | 26497 | 393593 |
| Coffee type: Instant coffee | -1.290911071 | 1.258087401 | 0.380354144 | MR-Egger | 5 | 0 | 185482 | 144189 |
| Coffee type: Ground coffee, including espresso, filter, etc. | 0.252851984 | 0.55885273 | 0.655375994 | MR-Egger | 24 | 0 | 73906 | 255765 |
| Alcohol usually taken with meals | 0.551404093 | 0.572033915 | 0.346589359 | MR-Egger | 22 | 0 | 143760 | 70463 |
| Spread type: Butter/spreadable butter | -1.585178622 | 0.953358843 | 0.120274628 | MR-Egger | 15 | 2 | 152410 | 267091 |
| Spread type: Never/rarely use spread | 1.598162982 | 1.212463334 | 0.318185113 | MR-Egger | 4 | 1 | 42617 | 376884 |
| Other fruit intake | -0.18186068 | 0.325550467 | 0.615402898 | MR-Egger | 5 | 1 | 58890 | - |
| Smoking status: Never | -0.15479207 | 0.044277302 | 0.000654425 | Weighted Median | 126 | 2 | 227144 | 191673 |
| Cereal type: Muesli | -0.274925488 | 0.091803711 | 0.011176028 | Weighted Median | 13 | 0 | 70931 | 278596 |
| Cereal type: Other, e.g. Cornflakes, Frosties | 0.116192936 | 0.12347181 | 0.374223235 | Weighted Median | 9 | 0 | 68111 | 281416 |
| Bread type: Wholemeal or wholegrain | 0.091315192 | 0.109724275 | 0.415620606 | Weighted Median | 20 | 1 | 233278 | 172429 |
| Bread type: White | 0.143075101 | 0.074444146 | 0.062559616 | Weighted Median | 37 | 2 | 107422 | 298285 |
| Seeds intake | 0.054994001 | 0.028689775 | 0.113385702 | Weighted Median | 6 | 0 | 58890 | - |
| Milk type used: Skimmed | 0.519413015 | 0.167580399 | 0.053316957 | Weighted Median | 4 | 1 | 86430 | 333660 |
| Milk type used: Full cream | 0.052325384 | 0.096644307 | 0.616962653 | Weighted Median | 5 | 0 | 26497 | 393593 |
| Coffee type: Instant coffee | -0.256300874 | 0.165327565 | 0.196014057 | Weighted Median | 5 | 0 | 185482 | 144189 |
| Coffee type: Ground coffee, including espresso, filter, etc. | -0.022591594 | 0.075618111 | 0.76780658 | Weighted Median | 24 | 0 | 73906 | 255765 |
| Alcohol usually taken with meals | -0.102739063 | 0.071918395 | 0.167839142 | Weighted Median | 22 | 0 | 143760 | 70463 |
| Spread type: Butter/spreadable butter | -0.085973422 | 0.154064081 | 0.585633218 | Weighted Median | 15 | 2 | 152410 | 267091 |
| Spread type: Never/rarely use spread | 0.150393198 | 0.178499517 | 0.46135345 | Weighted Median | 4 | 1 | 42617 | 376884 |
| Other fruit intake | -0.013790852 | 0.071897055 | 0.857231759 | Weighted Median | 5 | 1 | 58890 | - |
| Smoking status: Never | -0.155527 | 0.030818 | 1.57451E-06 | MR-PRESSO-IVW | 124 | 2 | 227144 | 191673 |
| Bread type: Wholemeal or wholegrain | -0.014197153 | 0.096438999 | 0.884599645 | MR-PRESSO-IVW | 19 | 1 | 233278 | 172429 |
| Bread type: White | 0.069004649 | 0.06707394 | 0.310844363 | MR-PRESSO-IVW | 35 | 2 | 107422 | 298285 |
| Milk type used: Skimmed | 0.338012081 | 0.243758289 | 0.299880914 | MR-PRESSO-IVW | 3 | 1 | 86430 | 333660 |
| Spread type: Butter/spreadable butter | 0.015087621 | 0.102387076 | 0.885295848 | MR-PRESSO-IVW | 13 | 2 | 152410 | 267091 |
| Spread type: Never/rarely use spread | 0.02460643 | 0.079927225 | 0.787291493 | MR-PRESSO-IVW | 3 | 1 | 42617 | 376884 |
| Other fruit intake | -0.01648073 | 0.03034978 | 0.62486 | MR-PRESSO-IVW | 4 | 1 | 58890 | - |

**Supplemental Table 2. Single-nucleotide polymorphisms (SNPs) instrumenting muesli intake as a nominal categorical trait (UKB data-field 1468) and their biological associations.**

| **SNP** | **Chr** | **Pos** | **Effect** | **Beta** | **Sd** | **Nearest Gene** | **Gene**  **Name** | **Associated Biology** |
| --- | --- | --- | --- | --- | --- | --- | --- | --- |
| rs10129747 | 14 | 77433198 | A | 0.009601 | 0.0095028 | IRF2BPL | Interferon regulatory factor 2 binding protein like | Female reproductive development, Neuronal maintenance |
| rs11514731 | 7 | 2051503 | C | 0.021894 | 0.0111593 | MAD1L1 | Mitotic arrest deficient 1 like 1; mitotic spindle assembly checkpoint protein MAD1; human accelerated region 3 | Cell cycle regulation |
| rs1271744 | 11 | 95594301 | G | 0.004586 | 0.0104011 | MTMR2 | Myotubularin related protein 2 | Lipid metabolism |
| rs13135092 | 4 | 103198082 | A | 0.005292 | 0.0214149 | SLC39A8 | Solute carrier family 39 member 8 | Zinc transport |
| rs17035173 | 2 | 68507833 | A | 0.004599 | 0.0106759 | CNRIP1 | Cannabinoid receptor interacting protein 1 | Receptor signaling modulation |
| rs4580876 | 6 | 98322872 | G | 0.006393 | 0.0093279 | MMS22L | MMS22 like, DNA repair protein | DNA damage repair |
| rs4877152 | 9 | 82438585 | G | 0.015918 | 0.0092247 | TLE4 | Transducin like enhancer of split 4 | N/A |
| rs62031562 | 16 | 28609329 | T | 0.014726 | 0.0106336 | SULT1A2 | Sulfotransferase family 1A member 2 | Lipid metabolism, Steroid metabolism, Catecholamine metabolism |
| rs73093445 | 3 | 71542279 | C | 0.026672 | 0.0099742 | FOXP1 | Forkhead box P1 | Development, Cell differentiation |
| rs7613360 | 3 | 49916710 | T | 0.031279 | 0.0102349 | MST1R | Macrophage stimulating 1 receptor | Immunity, Cell migration and proliferation |
| rs7806045 | 7 | 132610266 | C | 0.014318 | 0.0109519 | CHCHD3 | Coiled-coil-helix-coiled-coil-helix domain containing 3 | Mitochondrial structural integrity; Transcription regulation |
| rs7920624 | 10 | 67963186 | T | 0.009415 | 0.0092225 | CTNNA3 | Catenin alpha 3 | Cell adhesion |
| rs7932035 | 11 | 39620131 | A | -0.00192 | 0.012251 | LRRC4C | Leucine rich repeat containing 4C | Neuronal development |

**Supplemental Table 3. Causal estimates of muesli intake on CAD risk through 171 plasma metabolite levels.**

| **Metabolite** | **IVW_Beta** | **IVW_Sd** | **IVW_P** | **MRegger_Beta** | **MRegger_Sd** | **MRegger_P** | **Median_Beta** | **Median_Sd** | **Median_P** | **Unit** |
| --- | --- | --- | --- | --- | --- | --- | --- | --- | --- | --- |
| Unadjusted T/S ratio | -9.42E-01 | 4.99E-01 | 8.36E-02 | -1.90E+00 | 1.02E+00 | 8.85E-02 | -1.10E+00 | 6.07E-01 | 9.48E-02 | ratio |
| Adjusted T/S ratio | -9.68E-01 | 4.66E-01 | 6.00E-02 | -2.12E+00 | 9.23E-01 | 4.21E-02 | -1.50E+00 | 5.86E-01 | 2.53E-02 | ratio |
| Z-adjusted T/S log | -9.64E-01 | 4.67E-01 | 6.12E-02 | -2.10E+00 | 9.26E-01 | 4.41E-02 | -1.49E+00 | 5.87E-01 | 2.60E-02 | ratio |
| Total Cholesterol | -7.63E-01 | 7.23E-01 | 3.12E-01 | 3.68E-01 | 1.25E+00 | 7.74E-01 | 2.48E-01 | 8.17E-01 | 7.67E-01 | mmol/l |
| Total Cholesterol Minus HDL-C | 4.03E-02 | 8.67E-01 | 9.64E-01 | 2.45E+00 | 1.14E+00 | 5.41E-02 | 6.70E-01 | 9.70E-01 | 5.03E-01 | mmol/l |
| Remnant Cholesterol (Non-HDL, Non-LDL -Cholesterol) | 2.73E-01 | 8.36E-01 | 7.50E-01 | -1.37E+00 | 1.23E+00 | 2.88E-01 | 3.99E-01 | 9.12E-01 | 6.69E-01 | mmol/l |
| VLDL Cholesterol | 6.66E-01 | 6.06E-01 | 2.94E-01 | -1.03E+00 | 8.98E-01 | 2.76E-01 | 3.60E-01 | 6.25E-01 | 5.76E-01 | mmol/l |
| Clinical LDL Cholesterol | -1.40E-01 | 9.10E-01 | 8.80E-01 | 2.73E+00 | 1.25E+00 | 5.07E-02 | 8.04E-01 | 1.05E+00 | 4.60E-01 | mmol/l |
| LDL Cholesterol | -2.04E-01 | 8.80E-01 | 8.20E-01 | 2.46E+00 | 1.38E+00 | 1.03E-01 | 8.06E-01 | 9.78E-01 | 4.26E-01 | mmol/l |
| HDL Cholesterol | -7.54E-01 | 3.57E-01 | 5.62E-02 | -3.70E-02 | 3.68E-01 | 9.22E-01 | -4.13E-01 | 3.49E-01 | 2.60E-01 | mmol/l |
| Total Triglycerides | 7.96E-01 | 5.81E-01 | 1.96E-01 | -2.13E-01 | 9.10E-01 | 8.19E-01 | 3.67E-01 | 6.02E-01 | 5.53E-01 | mmol/l |
| Triglycerides in VLDL | 8.71E-01 | 6.00E-01 | 1.72E-01 | -7.58E-01 | 8.32E-01 | 3.82E-01 | 4.68E-01 | 6.49E-01 | 4.84E-01 | mmol/l |
| Triglycerides in LDL | 6.98E-01 | 5.16E-01 | 2.01E-01 | -5.28E-01 | 6.57E-01 | 4.39E-01 | 3.08E-01 | 5.08E-01 | 5.56E-01 | mmol/l |
| Triglycerides in HDL | 4.01E-01 | 6.29E-01 | 5.36E-01 | 1.07E-01 | 9.21E-01 | 9.10E-01 | 8.06E-02 | 6.68E-01 | 9.06E-01 | mmol/l |
| Total Phospholipids in Lipoprotein Particles | -7.05E-01 | 6.27E-01 | 2.83E-01 | -5.42E-02 | 9.04E-01 | 9.53E-01 | -1.09E-01 | 6.24E-01 | 8.64E-01 | mmol/l |
| Phospholipids in VLDL | 7.71E-01 | 5.64E-01 | 1.96E-01 | -4.51E-01 | 8.52E-01 | 6.07E-01 | 3.52E-01 | 5.90E-01 | 5.62E-01 | mmol/l |
| Phospholipids in LDL | 1.36E-01 | 9.10E-01 | 8.84E-01 | 3.00E+00 | 1.15E+00 | 2.40E-02 | 1.01E+00 | 1.01E+00 | 3.40E-01 | mmol/l |
| Phospholipids in HDL | -7.28E-01 | 3.89E-01 | 8.56E-02 | 8.03E-02 | 3.89E-01 | 8.40E-01 | -3.68E-01 | 3.51E-01 | 3.16E-01 | mmol/l |
| Total Esterified Cholesterol | -8.13E-01 | 6.97E-01 | 2.66E-01 | 9.55E-02 | 1.18E+00 | 9.37E-01 | 2.28E-01 | 8.01E-01 | 7.81E-01 | mmol/l |
| Cholesteryl Esters in VLDL | 5.91E-01 | 6.29E-01 | 3.66E-01 | -2.48E-01 | 1.11E+00 | 8.27E-01 | 3.53E-01 | 6.47E-01 | 5.95E-01 | mmol/l |
| Cholesteryl Esters in LDL | -6.13E-02 | 8.56E-01 | 9.44E-01 | 2.80E+00 | 1.22E+00 | 4.21E-02 | 8.97E-01 | 8.79E-01 | 3.27E-01 | mmol/l |
| Cholesteryl Esters in HDL | -7.59E-01 | 3.56E-01 | 5.43E-02 | -5.22E-02 | 3.67E-01 | 8.89E-01 | -4.47E-01 | 3.58E-01 | 2.36E-01 | mmol/l |
| Total Free Cholesterol | -5.63E-01 | 7.79E-01 | 4.83E-01 | 1.17E+00 | 1.29E+00 | 3.83E-01 | 3.22E-01 | 9.49E-01 | 7.40E-01 | mmol/l |
| Free Cholesterol in VLDL | 7.48E-01 | 5.85E-01 | 2.25E-01 | -6.93E-01 | 8.97E-01 | 4.56E-01 | 4.20E-01 | 5.99E-01 | 4.96E-01 | mmol/l |
| Free Cholesterol in LDL | -5.74E-01 | 9.11E-01 | 5.41E-01 | 1.98E+00 | 1.56E+00 | 2.31E-01 | 5.63E-01 | 1.12E+00 | 6.24E-01 | mmol/l |
| Free Cholesterol in HDL | -7.50E-01 | 3.68E-01 | 6.39E-02 | 3.36E-02 | 3.84E-01 | 9.32E-01 | -3.56E-01 | 3.79E-01 | 3.66E-01 | mmol/l |
| Total Lipids in Lipoprotein Particles | -2.95E-01 | 7.89E-01 | 7.15E-01 | 1.42E+00 | 1.10E+00 | 2.25E-01 | 3.45E-01 | 9.15E-01 | 7.13E-01 | mmol/l |
| Total Lipids in VLDL | 8.10E-01 | 5.79E-01 | 1.87E-01 | -8.16E-01 | 7.95E-01 | 3.27E-01 | 3.65E-01 | 5.94E-01 | 5.51E-01 | mmol/l |
| Total Lipids in LDL | 5.19E-03 | 8.77E-01 | 9.95E-01 | 2.80E+00 | 1.19E+00 | 3.86E-02 | 9.12E-01 | 9.20E-01 | 3.41E-01 | mmol/l |
| Total Lipids in HDL | -7.32E-01 | 3.75E-01 | 7.44E-02 | 5.03E-02 | 3.82E-01 | 8.98E-01 | -3.79E-01 | 3.52E-01 | 3.02E-01 | mmol/l |
| Total Concentration of Lipoprotein Particles | -7.19E-01 | 4.74E-01 | 1.55E-01 | -2.35E-01 | 5.67E-01 | 6.86E-01 | -4.65E-01 | 4.35E-01 | 3.06E-01 | mmol/l |
| Concentration of VLDL Particles | 6.92E-01 | 5.92E-01 | 2.65E-01 | -7.92E-01 | 8.71E-01 | 3.83E-01 | 3.45E-01 | 5.85E-01 | 5.67E-01 | mmol/l |
| Concentration of LDL Particles | 2.83E-01 | 8.17E-01 | 7.35E-01 | 2.01E+00 | 1.16E+00 | 1.12E-01 | 7.74E-01 | 8.72E-01 | 3.92E-01 | mmol/l |
| Concentration of HDL Particles | -7.12E-01 | 4.43E-01 | 1.34E-01 | -2.16E-01 | 5.07E-01 | 6.79E-01 | -4.78E-01 | 4.16E-01 | 2.72E-01 | mmol/l |
| Average Diameter for VLDL Particles | 9.37E-01 | 6.11E-01 | 1.51E-01 | 2.32E-02 | 9.60E-01 | 9.81E-01 | 5.82E-01 | 6.06E-01 | 3.56E-01 | nm |
| Average Diameter for LDL Particles | -2.39E+00 | 7.44E-01 | 7.44E-03 | -1.17E+00 | 1.29E+00 | 3.85E-01 | -1.82E+00 | 1.44E+00 | 2.30E-01 | nm |
| Average Diameter for HDL Particles | -7.46E-01 | 3.42E-01 | 4.99E-02 | 1.80E-01 | 3.54E-01 | 6.22E-01 | -4.34E-01 | 3.66E-01 | 2.59E-01 | nm |
| Phosphoglycerides | -7.78E-01 | 5.74E-01 | 2.00E-01 | -3.23E-01 | 8.07E-01 | 6.97E-01 | -2.99E-01 | 5.94E-01 | 6.24E-01 | mmol/l |
| Total Cholines | -8.00E-01 | 5.52E-01 | 1.73E-01 | -3.97E-01 | 7.85E-01 | 6.23E-01 | -3.50E-01 | 5.52E-01 | 5.38E-01 | mmol/l |
| Phosphatidylcholines | -8.89E-01 | 4.99E-01 | 1.00E-01 | -6.41E-01 | 7.11E-01 | 3.86E-01 | -9.44E-01 | 5.36E-01 | 1.04E-01 | mmol/l |
| Sphingomyelins | -4.96E-01 | 6.03E-01 | 4.27E-01 | 4.43E-01 | 7.21E-01 | 5.52E-01 | 1.33E-01 | 5.28E-01 | 8.05E-01 | mmol/l |
| Apolipoprotein B | 4.16E-01 | 8.09E-01 | 6.16E-01 | 7.90E-01 | 1.32E+00 | 5.62E-01 | 7.08E-01 | 8.07E-01 | 3.98E-01 | g/l |
| Apolipoprotein A1 | -7.30E-01 | 4.00E-01 | 9.29E-02 | 4.44E-02 | 3.86E-01 | 9.10E-01 | -5.82E-01 | 3.68E-01 | 1.39E-01 | g/l |
| Total Fatty Acids | 4.80E-01 | 7.39E-01 | 5.29E-01 | -2.85E-01 | 1.09E+00 | 7.98E-01 | 5.60E-01 | 7.17E-01 | 4.50E-01 | mmol/l |
| Degree of Unsaturation | -1.24E+00 | 4.75E-01 | 2.26E-02 | -7.81E-01 | 1.02E+00 | 4.58E-01 | -9.69E-01 | 5.89E-01 | 1.26E-01 | degree |
| Omega-3 Fatty Acids | -8.23E-01 | 6.44E-01 | 2.26E-01 | -2.18E+00 | 1.28E+00 | 1.16E-01 | -1.32E+00 | 7.68E-01 | 1.11E-01 | mmol/l |
| Omega-6 Fatty Acids | -5.84E-01 | 7.46E-01 | 4.48E-01 | -1.23E+00 | 1.33E+00 | 3.76E-01 | 2.74E-01 | 8.53E-01 | 7.54E-01 | mmol/l |
| Polyunsaturated Fatty Acids | -6.54E-01 | 7.04E-01 | 3.71E-01 | -1.91E+00 | 1.41E+00 | 2.02E-01 | 2.83E-01 | 8.37E-01 | 7.42E-01 | mmol/l |
| Monounsaturated Fatty Acids | 9.71E-01 | 5.55E-01 | 1.06E-01 | 1.59E-01 | 1.01E+00 | 8.78E-01 | 7.70E-01 | 5.82E-01 | 2.11E-01 | mmol/l |
| Saturated Fatty Acids | 6.30E-01 | 7.59E-01 | 4.23E-01 | -1.83E+00 | 8.04E-01 | 4.39E-02 | 4.25E-01 | 8.88E-01 | 6.41E-01 | mmol/l |
| Linoleic Acid | -9.17E-01 | 7.11E-01 | 2.22E-01 | -4.21E-01 | 1.16E+00 | 7.24E-01 | 8.13E-03 | 9.37E-01 | 9.93E-01 | mmol/l |
| Docosahexaenoic Acid | -9.97E-01 | 4.53E-01 | 4.79E-02 | -1.21E+00 | 9.20E-01 | 2.16E-01 | -1.00E+00 | 5.49E-01 | 9.39E-02 | mmol/l |
| Alanine | -7.97E-01 | 6.96E-01 | 2.75E-01 | -2.83E-01 | 1.22E+00 | 8.21E-01 | -1.19E+00 | 9.03E-01 | 2.10E-01 | mmol/l |
| Glutamine | -9.36E-01 | 4.07E-01 | 4.00E-02 | -1.54E-01 | 5.29E-01 | 7.76E-01 | -7.73E-01 | 4.93E-01 | 1.43E-01 | mmol/l |
| Glycine | -1.95E-01 | 6.07E-01 | 7.53E-01 | 1.93E-01 | 8.49E-01 | 8.24E-01 | -1.43E-01 | 5.23E-01 | 7.90E-01 | mmol/l |
| Histidine | -9.95E-01 | 5.88E-01 | 1.16E-01 | -2.20E+00 | 8.43E-01 | 2.40E-02 | -1.20E+00 | 6.97E-01 | 1.10E-01 | mmol/l |
| Total Concentration of Branched-Chain Amino Acids | 4.08E-01 | 7.72E-01 | 6.07E-01 | 5.16E-01 | 1.07E+00 | 6.40E-01 | 4.45E-01 | 7.68E-01 | 5.73E-01 | mmol/l |
| Isoleucine | 6.53E-01 | 9.13E-01 | 4.88E-01 | 3.66E-01 | 1.34E+00 | 7.90E-01 | 8.02E-01 | 1.10E+00 | 4.81E-01 | mmol/l |
| Leucine | 3.70E-01 | 7.36E-01 | 6.25E-01 | 6.29E-02 | 1.04E+00 | 9.53E-01 | 4.32E-01 | 7.85E-01 | 5.92E-01 | mmol/l |
| Valine | 3.30E-01 | 7.82E-01 | 6.81E-01 | 1.08E+00 | 1.06E+00 | 3.32E-01 | 4.07E-01 | 7.73E-01 | 6.08E-01 | mmol/l |
| Phenylalanine | 2.60E-01 | 8.25E-01 | 7.58E-01 | -9.86E-01 | 1.15E+00 | 4.09E-01 | -1.20E-01 | 9.03E-01 | 8.97E-01 | mmol/l |
| Tyrosine | -2.85E-01 | 6.11E-01 | 6.49E-01 | -5.00E-01 | 8.70E-01 | 5.77E-01 | -4.32E-02 | 5.55E-01 | 9.39E-01 | mmol/l |
| Glucose | 1.36E+00 | 9.55E-01 | 1.79E-01 | 1.17E+00 | 1.92E+00 | 5.55E-01 | 1.19E+00 | 1.25E+00 | 3.61E-01 | mmol/l |
| Lactate | 1.08E+00 | 1.20E+00 | 3.85E-01 | 2.72E-01 | 1.85E+00 | 8.86E-01 | 1.05E+00 | 4.54E+00 | 8.20E-01 | mmol/l |
| Pyruvate | -1.02E+00 | 8.11E-01 | 2.33E-01 | -2.07E+00 | 1.09E+00 | 8.31E-02 | -1.15E+00 | 1.20E+00 | 3.54E-01 | mmol/l |
| Citrate | -7.80E-02 | 9.29E-01 | 9.34E-01 | -6.98E-01 | 1.37E+00 | 6.20E-01 | 8.12E-01 | 1.52E+00 | 6.03E-01 | mmol/l |
| 3-Hydroxybutyrate | 1.06E+00 | 6.17E-01 | 1.11E-01 | 2.67E-01 | 8.72E-01 | 7.65E-01 | 1.12E+00 | 6.26E-01 | 9.96E-02 | mmol/l |
| Acetate | -3.29E+00 | 5.88E-01 | 1.15E-04 | -2.87E+00 | 9.98E-01 | 1.50E-02 | -3.49E+00 | 1.82E+00 | 7.96E-02 | mmol/l |
| Acetoacetate | 4.16E-01 | 9.40E-01 | 6.66E-01 | 1.19E+00 | 1.25E+00 | 3.63E-01 | 5.39E-01 | 1.26E+00 | 6.77E-01 | mmol/l |
| Acetone | -1.31E+00 | 7.53E-01 | 1.07E-01 | -2.51E-01 | 1.73E+00 | 8.87E-01 | -1.30E+00 | 1.23E+00 | 3.11E-01 | mmol/l |
| Creatinine | 1.17E+00 | 8.05E-01 | 1.71E-01 | 9.78E-02 | 1.18E+00 | 9.35E-01 | 1.76E+00 | 1.05E+00 | 1.21E-01 | mmol/l |
| Albumin | -5.45E-01 | 5.52E-01 | 3.43E-01 | 3.91E-01 | 7.96E-01 | 6.33E-01 | 2.02E-01 | 5.56E-01 | 7.23E-01 | g/l |
| Glycoprotein Acetyls | 1.15E+00 | 2.51E-01 | 6.41E-04 | 3.59E-01 | 4.95E-01 | 4.82E-01 | 7.63E-01 | 4.14E-01 | 8.99E-02 | mmol/l |
| Concentration of Chylomicrons and Extremely Large VLDL Particles | 9.64E-01 | 5.48E-01 | 1.04E-01 | 2.14E-01 | 9.56E-01 | 8.27E-01 | 4.77E-01 | 5.69E-01 | 4.19E-01 | mmol/l |
| Total Lipids in Chylomicrons and Extremely Large VLDL | 1.02E+00 | 5.81E-01 | 1.03E-01 | 2.25E-01 | 1.02E+00 | 8.30E-01 | 4.98E-01 | 6.46E-01 | 4.56E-01 | mmol/l |
| Phospholipids in Chylomicrons and Extremely Large VLDL | 9.25E-01 | 5.22E-01 | 1.02E-01 | 2.17E-01 | 9.15E-01 | 8.17E-01 | 4.37E-01 | 5.65E-01 | 4.55E-01 | mmol/l |
| Cholesterol in Chylomicrons and Extremely Large VLDL | 7.99E-01 | 4.89E-01 | 1.28E-01 | -4.14E-01 | 7.02E-01 | 5.67E-01 | 3.37E-01 | 4.97E-01 | 5.11E-01 | mmol/l |
| Cholesteryl Esters in Chylomicrons and Extremely Large VLDL | 7.77E-01 | 4.62E-01 | 1.18E-01 | -2.93E-01 | 6.56E-01 | 6.64E-01 | 3.17E-01 | 4.64E-01 | 5.07E-01 | mmol/l |
| Free Cholesterol in Chylomicrons and Extremely Large VLDL | 8.58E-01 | 5.43E-01 | 1.40E-01 | -5.88E-01 | 7.87E-01 | 4.70E-01 | 4.09E-01 | 5.48E-01 | 4.70E-01 | mmol/l |
| Triglycerides in Chylomicrons and Extremely Large VLDL | 1.14E+00 | 6.53E-01 | 1.08E-01 | 1.14E-01 | 1.24E+00 | 9.29E-01 | 7.66E-01 | 7.88E-01 | 3.50E-01 | mmol/l |
| Concentration of Very Large VLDL Particles | 9.10E-01 | 5.25E-01 | 1.08E-01 | -3.69E-01 | 7.79E-01 | 6.45E-01 | 4.82E-01 | 5.40E-01 | 3.89E-01 | mmol/l |
| Total Lipids in Very Large VLDL | 9.24E-01 | 5.39E-01 | 1.12E-01 | -4.05E-01 | 7.97E-01 | 6.21E-01 | 4.74E-01 | 5.26E-01 | 3.85E-01 | mmol/l |
| Phospholipids in Very Large VLDL | 9.26E-01 | 5.20E-01 | 1.00E-01 | -3.30E-01 | 7.83E-01 | 6.82E-01 | 4.60E-01 | 5.36E-01 | 4.08E-01 | mmol/l |
| Cholesterol in Very Large VLDL | 8.31E-01 | 5.02E-01 | 1.24E-01 | -3.87E-01 | 7.20E-01 | 6.02E-01 | 3.95E-01 | 4.96E-01 | 4.41E-01 | mmol/l |
| Cholesteryl Esters in Very Large VLDL | 7.71E-01 | 4.90E-01 | 1.41E-01 | -3.73E-01 | 6.70E-01 | 5.89E-01 | 3.73E-01 | 4.69E-01 | 4.43E-01 | mmol/l |
| Free Cholesterol in Very Large VLDL | 8.95E-01 | 5.31E-01 | 1.18E-01 | -4.25E-01 | 7.80E-01 | 5.96E-01 | 4.21E-01 | 5.21E-01 | 4.35E-01 | mmol/l |
| Triglycerides in Very Large VLDL | 9.36E-01 | 5.73E-01 | 1.28E-01 | -4.98E-01 | 8.26E-01 | 5.59E-01 | 5.36E-01 | 5.77E-01 | 3.71E-01 | mmol/l |
| Concentration of Large VLDL Particles | 8.54E-01 | 5.50E-01 | 1.46E-01 | -5.91E-01 | 7.84E-01 | 4.67E-01 | 4.99E-01 | 5.44E-01 | 3.77E-01 | mmol/l |
| Total Lipids in Large VLDL | 8.61E-01 | 5.88E-01 | 1.69E-01 | -7.38E-01 | 8.20E-01 | 3.87E-01 | 5.81E-01 | 5.86E-01 | 3.41E-01 | mmol/l |
| Phospholipids in Large VLDL | 8.48E-01 | 5.25E-01 | 1.33E-01 | -4.30E-01 | 7.42E-01 | 5.74E-01 | 4.36E-01 | 4.97E-01 | 3.98E-01 | mmol/l |
| Cholesterol in Large VLDL | 7.89E-01 | 5.38E-01 | 1.68E-01 | -5.85E-01 | 7.34E-01 | 4.42E-01 | 4.21E-01 | 5.44E-01 | 4.54E-01 | mmol/l |
| Cholesteryl Esters in Large VLDL | 7.15E-01 | 5.62E-01 | 2.28E-01 | -4.74E-01 | 8.07E-01 | 5.69E-01 | 3.93E-01 | 5.43E-01 | 4.82E-01 | mmol/l |
| Free Cholesterol in Large VLDL | 8.44E-01 | 5.20E-01 | 1.30E-01 | -4.63E-01 | 7.49E-01 | 5.49E-01 | 4.72E-01 | 5.21E-01 | 3.84E-01 | mmol/l |
| Triglycerides in Large VLDL | 8.57E-01 | 6.66E-01 | 2.23E-01 | -1.06E+00 | 8.80E-01 | 2.55E-01 | 8.51E-01 | 6.62E-01 | 2.23E-01 | mmol/l |
| Concentration of Medium VLDL Particles | 6.01E-01 | 6.76E-01 | 3.92E-01 | -1.10E+00 | 8.86E-01 | 2.42E-01 | 6.77E-01 | 6.69E-01 | 3.32E-01 | mmol/l |
| Total Lipids in Medium VLDL | 5.85E-01 | 6.76E-01 | 4.04E-01 | 3.27E-01 | 1.07E+00 | 7.65E-01 | 3.74E-01 | 7.14E-01 | 6.10E-01 | mmol/l |
| Phospholipids in Medium VLDL | 5.44E-01 | 6.95E-01 | 4.49E-01 | -1.32E+00 | 8.87E-01 | 1.66E-01 | 5.39E-01 | 6.97E-01 | 4.55E-01 | mmol/l |
| Cholesterol in Medium VLDL | 1.54E-01 | 8.28E-01 | 8.55E-01 | 1.99E+00 | 1.27E+00 | 1.46E-01 | 5.57E-01 | 8.66E-01 | 5.32E-01 | mmol/l |
| Cholesteryl Esters in Medium VLDL | -1.84E-01 | 8.66E-01 | 8.36E-01 | 1.30E+00 | 1.35E+00 | 3.57E-01 | 4.81E-01 | 9.41E-01 | 6.18E-01 | mmol/l |
| Free Cholesterol in Medium VLDL | 4.53E-01 | 7.42E-01 | 5.52E-01 | -1.06E-01 | 1.21E+00 | 9.32E-01 | 5.94E-01 | 7.33E-01 | 4.34E-01 | mmol/l |
| Triglycerides in Medium VLDL | 6.96E-01 | 6.41E-01 | 2.99E-01 | -1.07E+00 | 7.72E-01 | 1.94E-01 | 5.52E-01 | 6.18E-01 | 3.90E-01 | mmol/l |
| Concentration of Small VLDL Particles | 6.70E-01 | 5.49E-01 | 2.46E-01 | -4.19E-01 | 7.53E-01 | 5.89E-01 | 3.34E-01 | 5.28E-01 | 5.40E-01 | mmol/l |
| Total Lipids in Small VLDL | 6.46E-01 | 5.64E-01 | 2.74E-01 | -5.51E-01 | 7.72E-01 | 4.90E-01 | 3.44E-01 | 5.36E-01 | 5.33E-01 | mmol/l |
| Phospholipids in Small VLDL | 5.80E-01 | 6.19E-01 | 3.67E-01 | -8.95E-01 | 8.14E-01 | 2.95E-01 | 5.23E-01 | 5.78E-01 | 3.84E-01 | mmol/l |
| Cholesterol in Small VLDL | 6.17E-01 | 5.73E-01 | 3.03E-01 | 7.68E-02 | 9.30E-01 | 9.36E-01 | 4.43E-01 | 5.81E-01 | 4.61E-01 | mmol/l |
| Cholesteryl Esters in Small VLDL | 6.48E-01 | 5.22E-01 | 2.38E-01 | 2.74E-01 | 8.41E-01 | 7.51E-01 | 3.61E-01 | 4.89E-01 | 4.74E-01 | mmol/l |
| Free Cholesterol in Small VLDL | 4.76E-01 | 6.88E-01 | 5.02E-01 | -4.90E-01 | 1.06E+00 | 6.52E-01 | 6.29E-01 | 6.58E-01 | 3.58E-01 | mmol/l |
| Triglycerides in Small VLDL | 6.81E-01 | 5.93E-01 | 2.73E-01 | -7.73E-01 | 7.11E-01 | 3.00E-01 | 3.04E-01 | 5.65E-01 | 6.01E-01 | mmol/l |
| Concentration of Very Small VLDL Particles | 5.77E-01 | 6.87E-01 | 4.17E-01 | -1.21E+00 | 9.89E-01 | 2.45E-01 | 3.56E-01 | 7.88E-01 | 6.59E-01 | mmol/l |
| Total Lipids in Very Small VLDL | 4.62E-01 | 7.02E-01 | 5.23E-01 | -1.61E+00 | 9.60E-01 | 1.22E-01 | 3.23E-01 | 7.99E-01 | 6.93E-01 | mmol/l |
| Phospholipids in Very Small VLDL | 6.00E-01 | 6.50E-01 | 3.74E-01 | -1.47E+00 | 9.20E-01 | 1.37E-01 | 3.07E-01 | 6.92E-01 | 6.65E-01 | mmol/l |
| Cholesterol in Very Small VLDL | -1.62E-02 | 7.81E-01 | 9.84E-01 | -1.27E+00 | 1.27E+00 | 3.41E-01 | -1.64E-01 | 8.86E-01 | 8.56E-01 | mmol/l |
| Cholesteryl Esters in Very Small VLDL | -2.60E-01 | 7.74E-01 | 7.43E-01 | -1.98E+00 | 1.17E+00 | 1.18E-01 | -5.76E-01 | 8.56E-01 | 5.14E-01 | mmol/l |
| Free Cholesterol in Very Small VLDL | 4.87E-01 | 7.15E-01 | 5.08E-01 | -1.70E+00 | 9.97E-01 | 1.16E-01 | 3.31E-01 | 7.89E-01 | 6.83E-01 | mmol/l |
| Triglycerides in Very Small VLDL | 6.58E-01 | 5.31E-01 | 2.39E-01 | -4.74E-01 | 6.29E-01 | 4.67E-01 | 1.97E-01 | 4.94E-01 | 6.97E-01 | mmol/l |
| Concentration of IDL Particles | 4.88E-01 | 9.38E-01 | 6.12E-01 | -1.13E+00 | 1.61E+00 | 4.98E-01 | 5.57E-01 | 1.14E+00 | 6.34E-01 | mmol/l |
| Total Lipids in IDL | -6.45E-01 | 8.27E-01 | 4.50E-01 | 1.63E+00 | 1.15E+00 | 1.86E-01 | 3.18E-01 | 9.15E-01 | 7.35E-01 | mmol/l |
| Phospholipids in IDL | -5.98E-01 | 7.97E-01 | 4.68E-01 | 1.54E+00 | 1.09E+00 | 1.86E-01 | 7.08E-02 | 8.93E-01 | 9.38E-01 | mmol/l |
| Cholesterol in IDL | -7.82E-01 | 7.95E-01 | 3.45E-01 | 1.15E+00 | 1.17E+00 | 3.45E-01 | 2.04E-01 | 8.30E-01 | 8.10E-01 | mmol/l |
| Cholesteryl Esters in IDL | -8.58E-01 | 7.69E-01 | 2.86E-01 | 8.48E-01 | 1.16E+00 | 4.78E-01 | -1.13E-01 | 8.36E-01 | 8.95E-01 | mmol/l |
| Free Cholesterol in IDL | -4.74E-01 | 8.68E-01 | 5.95E-01 | 1.94E+00 | 1.09E+00 | 1.04E-01 | 3.96E-01 | 8.97E-01 | 6.67E-01 | mmol/l |
| Triglycerides in IDL | 6.61E-01 | 5.43E-01 | 2.46E-01 | -2.68E-01 | 7.01E-01 | 7.09E-01 | 2.24E-01 | 5.45E-01 | 6.89E-01 | mmol/l |
| Concentration of Large LDL Particles | -1.24E-02 | 8.57E-01 | 9.89E-01 | 2.89E+00 | 1.18E+00 | 3.26E-02 | 6.18E-01 | 9.89E-01 | 5.44E-01 | mmol/l |
| Total Lipids in Large LDL | -3.66E-01 | 8.79E-01 | 6.85E-01 | 1.95E+00 | 1.47E+00 | 2.09E-01 | 6.23E-01 | 1.05E+00 | 5.63E-01 | mmol/l |
| Phospholipids in Large LDL | -1.40E-01 | 8.83E-01 | 8.76E-01 | 2.28E+00 | 1.30E+00 | 1.08E-01 | 8.65E-01 | 9.83E-01 | 3.96E-01 | mmol/l |
| Cholesterol in Large LDL | -5.94E-01 | 8.67E-01 | 5.07E-01 | 1.91E+00 | 1.55E+00 | 2.43E-01 | 5.16E-01 | 9.99E-01 | 6.15E-01 | mmol/l |
| Cholesteryl Esters in Large LDL | -5.34E-01 | 8.72E-01 | 5.52E-01 | 1.75E+00 | 1.63E+00 | 3.05E-01 | 5.50E-01 | 1.09E+00 | 6.23E-01 | mmol/l |
| Free Cholesterol in Large LDL | -7.08E-01 | 8.46E-01 | 4.19E-01 | 1.12E+00 | 1.48E+00 | 4.65E-01 | 4.15E-01 | 9.85E-01 | 6.81E-01 | mmol/l |
| Triglycerides in Large LDL | 7.19E-01 | 5.29E-01 | 1.99E-01 | -6.05E-02 | 7.82E-01 | 9.40E-01 | 3.20E-01 | 5.42E-01 | 5.65E-01 | mmol/l |
| Concentration of Medium LDL Particles | 6.30E-01 | 7.58E-01 | 4.22E-01 | 3.05E-01 | 1.23E+00 | 8.08E-01 | 9.20E-01 | 7.60E-01 | 2.50E-01 | mmol/l |
| Total Lipids in Medium LDL | 5.00E-01 | 7.62E-01 | 5.24E-01 | 2.58E+00 | 8.83E-01 | 1.38E-02 | 1.19E+00 | 7.52E-01 | 1.40E-01 | mmol/l |
| Phospholipids in Medium LDL | 5.41E-01 | 7.94E-01 | 5.09E-01 | 2.89E+00 | 9.05E-01 | 8.61E-03 | 1.23E+00 | 8.29E-01 | 1.64E-01 | mmol/l |
| Cholesterol in Medium LDL | 4.18E-01 | 7.75E-01 | 5.99E-01 | 2.45E+00 | 9.45E-01 | 2.50E-02 | 1.13E+00 | 7.87E-01 | 1.77E-01 | mmol/l |
| Cholesteryl Esters in Medium LDL | 5.10E-01 | 7.22E-01 | 4.94E-01 | 9.78E-01 | 1.11E+00 | 3.96E-01 | 1.15E+00 | 7.03E-01 | 1.28E-01 | mmol/l |
| Free Cholesterol in Medium LDL | -8.82E-02 | 9.24E-01 | 9.26E-01 | 2.31E+00 | 1.31E+00 | 1.05E-01 | 9.23E-01 | 1.09E+00 | 4.14E-01 | mmol/l |
| Triglycerides in Medium LDL | 6.86E-01 | 5.06E-01 | 2.00E-01 | -5.16E-01 | 6.45E-01 | 4.40E-01 | 3.02E-01 | 4.89E-01 | 5.48E-01 | mmol/l |
| Concentration of Small LDL Particles | 6.73E-01 | 7.78E-01 | 4.04E-01 | 3.70E-01 | 1.28E+00 | 7.78E-01 | 6.75E-01 | 7.41E-01 | 3.80E-01 | mmol/l |
| Total Lipids in Small LDL | 5.06E-01 | 8.40E-01 | 5.58E-01 | 6.37E-01 | 1.35E+00 | 6.46E-01 | 1.01E+00 | 9.10E-01 | 2.87E-01 | mmol/l |
| Phospholipids in Small LDL | 3.92E-01 | 1.01E+00 | 7.06E-01 | -1.01E+00 | 1.79E+00 | 5.83E-01 | 1.28E+00 | 1.31E+00 | 3.49E-01 | mmol/l |
| Cholesterol in Small LDL | 4.15E-01 | 8.18E-01 | 6.21E-01 | 1.86E+00 | 1.16E+00 | 1.38E-01 | 1.14E+00 | 9.19E-01 | 2.37E-01 | mmol/l |
| Cholesteryl Esters in Small LDL | 5.09E-01 | 7.39E-01 | 5.04E-01 | 9.28E-01 | 1.13E+00 | 4.29E-01 | 8.54E-01 | 8.44E-01 | 3.32E-01 | mmol/l |
| Free Cholesterol in Small LDL | -2.12E-01 | 1.06E+00 | 8.44E-01 | 1.25E+00 | 1.70E+00 | 4.79E-01 | 1.08E+00 | 2.04E+00 | 6.06E-01 | mmol/l |
| Triglycerides in Small LDL | 6.90E-01 | 5.39E-01 | 2.25E-01 | -2.39E-01 | 8.05E-01 | 7.72E-01 | 2.97E-01 | 5.49E-01 | 5.98E-01 | mmol/l |
| Concentration of Very Large HDL Particles | -7.54E-01 | 3.84E-01 | 7.27E-02 | -8.60E-02 | 5.64E-01 | 8.82E-01 | -4.86E-01 | 4.22E-01 | 2.72E-01 | mmol/l |
| Total Lipids in Very Large HDL | -7.41E-01 | 3.77E-01 | 7.29E-02 | -3.68E-02 | 5.80E-01 | 9.50E-01 | -4.63E-01 | 4.11E-01 | 2.82E-01 | mmol/l |
| Phospholipids in Very Large HDL | -7.23E-01 | 3.72E-01 | 7.60E-02 | -2.41E-02 | 5.69E-01 | 9.67E-01 | -4.52E-01 | 3.78E-01 | 2.55E-01 | mmol/l |
| Cholesterol in Very Large HDL | -7.78E-01 | 3.85E-01 | 6.60E-02 | -7.16E-02 | 6.01E-01 | 9.07E-01 | -5.00E-01 | 4.23E-01 | 2.60E-01 | mmol/l |
| Cholesteryl Esters in Very Large HDL | -7.91E-01 | 3.70E-01 | 5.37E-02 | -1.71E-01 | 5.74E-01 | 7.72E-01 | -5.14E-01 | 3.97E-01 | 2.19E-01 | mmol/l |
| Free Cholesterol in Very Large HDL | -7.36E-01 | 4.59E-01 | 1.35E-01 | 4.03E-01 | 7.03E-01 | 5.78E-01 | -4.85E-01 | 4.49E-01 | 3.00E-01 | mmol/l |
| Triglycerides in Very Large HDL | -2.09E-01 | 7.25E-01 | 7.78E-01 | -5.60E-01 | 1.21E+00 | 6.53E-01 | -8.58E-01 | 7.97E-01 | 3.03E-01 | mmol/l |
| Concentration of Large HDL Particles | -7.41E-01 | 3.35E-01 | 4.72E-02 | 7.84E-02 | 3.36E-01 | 8.20E-01 | -4.44E-01 | 3.45E-01 | 2.22E-01 | mmol/l |
| Total Lipids in Large HDL | -7.27E-01 | 3.38E-01 | 5.24E-02 | 1.13E-01 | 3.36E-01 | 7.44E-01 | -3.73E-01 | 3.33E-01 | 2.85E-01 | mmol/l |
| Phospholipids in Large HDL | -7.14E-01 | 3.41E-01 | 5.83E-02 | 1.16E-01 | 3.32E-01 | 7.34E-01 | -3.20E-01 | 3.43E-01 | 3.69E-01 | mmol/l |
| Cholesterol in Large HDL | -7.69E-01 | 3.40E-01 | 4.30E-02 | 1.05E-01 | 3.52E-01 | 7.72E-01 | -4.70E-01 | 3.73E-01 | 2.31E-01 | mmol/l |
| Cholesteryl Esters in Large HDL | -7.84E-01 | 3.40E-01 | 4.00E-02 | 9.05E-02 | 3.56E-01 | 8.04E-01 | -4.97E-01 | 3.81E-01 | 2.17E-01 | mmol/l |
| Free Cholesterol in Large HDL | -7.33E-01 | 3.44E-01 | 5.44E-02 | -2.35E-01 | 4.76E-01 | 6.31E-01 | -4.12E-01 | 3.70E-01 | 2.87E-01 | mmol/l |
| Triglycerides in Large HDL | -5.54E-01 | 5.51E-01 | 3.35E-01 | -1.57E-01 | 7.30E-01 | 8.34E-01 | -9.95E-01 | 5.74E-01 | 1.09E-01 | mmol/l |
| Concentration of Medium HDL Particles | -7.24E-01 | 4.02E-01 | 9.70E-02 | 3.22E-02 | 3.82E-01 | 9.34E-01 | -6.37E-01 | 3.53E-01 | 9.60E-02 | mmol/l |
| Total Lipids in Medium HDL | -7.27E-01 | 4.19E-01 | 1.08E-01 | 4.17E-02 | 3.90E-01 | 9.17E-01 | -6.06E-01 | 3.72E-01 | 1.29E-01 | mmol/l |
| Phospholipids in Medium HDL | -7.28E-01 | 4.42E-01 | 1.25E-01 | -1.03E-01 | 4.77E-01 | 8.32E-01 | -5.16E-01 | 4.03E-01 | 2.25E-01 | mmol/l |
| Cholesterol in Medium HDL | -7.25E-01 | 3.90E-01 | 8.81E-02 | -2.36E-02 | 3.68E-01 | 9.50E-01 | -5.36E-01 | 3.55E-01 | 1.57E-01 | mmol/l |
| Cholesteryl Esters in Medium HDL | -7.21E-01 | 3.92E-01 | 9.10E-02 | -1.32E-01 | 4.15E-01 | 7.57E-01 | -5.28E-01 | 3.61E-01 | 1.70E-01 | mmol/l |
| Free Cholesterol in Medium HDL | -7.38E-01 | 3.89E-01 | 8.20E-02 | 1.77E-02 | 3.81E-01 | 9.64E-01 | -4.28E-01 | 3.64E-01 | 2.63E-01 | mmol/l |
| Triglycerides in Medium HDL | 3.88E-01 | 6.21E-01 | 5.44E-01 | 6.62E-02 | 9.53E-01 | 9.46E-01 | 5.20E-02 | 6.95E-01 | 9.42E-01 | mmol/l |
| Concentration of Small HDL Particles | 2.63E-02 | 6.31E-01 | 9.67E-01 | 2.32E+00 | 1.06E+00 | 5.03E-02 | 6.07E-01 | 6.40E-01 | 3.62E-01 | mmol/l |
| Total Lipids in Small HDL | -4.44E-02 | 6.68E-01 | 9.48E-01 | 1.72E+00 | 1.10E+00 | 1.47E-01 | 4.58E-01 | 6.72E-01 | 5.08E-01 | mmol/l |
| Phospholipids in Small HDL | -1.76E-01 | 6.68E-01 | 7.97E-01 | -5.99E-01 | 1.12E+00 | 6.03E-01 | 3.06E-01 | 6.48E-01 | 6.46E-01 | mmol/l |
| Cholesterol in Small HDL | -1.47E-01 | 6.19E-01 | 8.16E-01 | 1.55E+00 | 1.14E+00 | 2.04E-01 | 4.77E-01 | 5.77E-01 | 4.24E-01 | mmol/l |
| Cholesteryl Esters in Small HDL | -3.50E-02 | 6.07E-01 | 9.55E-01 | 1.66E+00 | 1.01E+00 | 1.30E-01 | 6.00E-01 | 5.73E-01 | 3.15E-01 | mmol/l |
| Free Cholesterol in Small HDL | -5.18E-01 | 6.66E-01 | 4.52E-01 | 1.09E-01 | 1.17E+00 | 9.27E-01 | 2.34E-01 | 7.09E-01 | 7.47E-01 | mmol/l |
| Triglycerides in Small HDL | 8.09E-01 | 4.94E-01 | 1.28E-01 | -3.53E-01 | 6.94E-01 | 6.21E-01 | 3.88E-01 | 5.20E-01 | 4.70E-01 | mmol/l |

**Supplemental Table 4. Causal estimates of muesli intake on CAD risk *through* muesli ingredients.**

| **Trait** | **UKB Code** | **Estimate** | **SD** | **P** | **Method** |
| --- | --- | --- | --- | --- | --- |
| Unsalted nuts intake | 102440 | -2.486314260 | 0.924546291 | 0.019698007 | IVW |
| Dried fruit added to cereal | 100880 | -0.475770981 | 0.142266981 | 0.005842378 | IVW |
| Cholesterol-lowering milk | 100920 | -0.067383457 | 0.033367444 | 0.066350482 | IVW |
| Fresh fruit consumers | 1309 | -3.496449276 | 1.816016410 | 0.078212136 | IVW |
| Seeds intake | 102450 | -0.625422972 | 1.998944354 | 0.759745195 | IVW |
| Oat cakes intake | 101260 | -0.550897942 | 1.109258192 | 0.628418353 | IVW |

**Supplemental Table 5. Causal estimates of muesli intake as an ordinal categorical trait (UKB data-field 100800) on CAD risk.**

| **Trait** | **UKB Code** | **Estimate** | **SD** | **P** | **Method** |
| --- | --- | --- | --- | --- | --- |
| Muesli intake | 100800 | -1.238818392 | 0.539284823 | 0.040405426 | IVW |
| Muesli intake | 100800 | -0.104796465 | 0.743124209 | 0.89040236 | Egger |
| Muesli intake | 100800 | -0.505810485 | 0.596035425 | 0.412698743 | Median |

**Supplemental Table 6. Multivariable causal estimates of muesli intake on CAD risk while accounting for acetate and/or LDL cholesterol.**

| **Trait** | **Estimate** | **SD** | **P** | **Method** |
| --- | --- | --- | --- | --- |
| Muesli accounting for LDL cholesterol | -0.320416932 | 0.062506981 | 0.0003303130 | IVW |
| Muesli accounting for acetate | -0.154822774 | 0.066751823 | 0.0406222286 | IVW |
| Muesli accounting for LDL cholesterol and acetate | -0.180080226 | 0.064301024 | 0.0187758255 | IVW |
